# Useful Items

**Published:** 1879-09

**Authors:** 


### USEFUL ITEMS.

Sixty drops, one teaspoonful, or drachm.
Four teaspoonfuls, one tablespoonful.
Four tablespoonfuls, one ounce.
Sixteen ounces, one pint, or pound.
Four ounces, one gill.
Two gills make half a pint.
Two pints make one quart.
A common tumbler holds half a pint.
One wine-glass is half a gill.
A teacup is one gill.

